# Real-time dengue forecast for outbreak alerts in Southern Taiwan

**DOI:** 10.1371/journal.pntd.0008434

**Published:** 2020-07-27

**Authors:** Yu-Chieh Cheng, Fang-Jing Lee, Ya-Ting Hsu, Eric V. Slud, Chao A. Hsiung, Chun-Hong Chen, Ching-Len Liao, Tzai-Hung Wen, Chiu-Wen Chang, Jui-Hun Chang, Hsiao-Yu Wu, Te-Pin Chang, Pei-Sheng Lin, Hui-Pin Ho, Wen-Feng Hung, Jing-Dong Chou, Hsiao-Hui Tsou

**Affiliations:** 1 Division of Biostatistics and Bioinformatics, Institute of Population Health Sciences, National Health Research Institutes, Miaoli County, Taiwan; 2 National Mosquito-Borne Diseases Control Research Center, National Health Research Institutes, Miaoli County, Taiwan; 3 Department of Mathematics, University of Maryland, College Park, Maryland, United States of America; 4 National Institute of Infectious Diseases and Vaccinology, National Health Research Institutes, Miaoli County, Taiwan; 5 Department of Geography, National Taiwan University, Taipei, Taiwan; 6 Department of Health, Kaohsiung City Government, Kaohsiung City, Taiwan; 7 Environmental Protection Bureau, Kaohsiung City Government, Kaohsiung City, Taiwan; 8 Soil and groundwater pollution remediation center, CPC Corporation, Taiwan; 9 Graduate Institute of Biostatistics, College of Public Health, China Medical University, Taichung, Taiwan; Yale School of Public Health, UNITED STATES

## Abstract

Dengue fever is a viral disease transmitted by mosquitoes. In recent decades, dengue fever has spread throughout the world. In 2014 and 2015, southern Taiwan experienced its most serious dengue outbreak in recent years. Some statistical models have been established in the past, however, these models may not be suitable for predicting huge outbreaks in 2014 and 2015. The control of dengue fever has become the primary task of local health agencies. This study attempts to predict the occurrence of dengue fever in order to achieve the purpose of timely warning. We applied a newly developed autoregressive model (AR model) to assess the association between daily weather variability and daily dengue case number in 2014 and 2015 in Kaohsiung, the largest city in southern Taiwan. This model also contained additional lagged weather predictors, and developed 5-day-ahead and 15-day-ahead predictive models. Our results indicate that numbers of dengue cases in Kaohsiung are associated with humidity and the biting rate (BR). Our model is simple, intuitive and easy to use. The developed model can be embedded in a "real-time" schedule, and the data (at present) can be updated daily or weekly based on the needs of public health workers. In this study, a simple model using only meteorological factors performed well. The proposed real-time forecast model can help health agencies take public health actions to mitigate the influences of the epidemic.

## Author summary

Meteorological conditions are the most frequently mentioned factors in the study of dengue fever. Some of the main factors other than the purely meteorological about which the public-health authorities might have data, such as numbers of cases or other current measurements of dengue outbreaks in neighboring cities, had been used in some of the past dengue studies. In this study, we developed models for predicting dengue case number based on past dengue case data and meteorological data. The goal of the models is to provide early warning of the occurrence of dengue fever to assist public health agencies in preparing an epidemic response plan.

## Introduction

Dengue is a mosquito-borne viral disease that is found in tropical and sub-tropical climates around the world [1]. In recent decades, dengue has spread globally due to population growth, urbanization and globalization, climate change, and a lack of effective vector control [2–4]. According to the World Health Organization (WHO), about half of the world’s population is at risk of infection with dengue viruses. It is estimated that there are 390 million dengue infections each year [1, 5, 6].

Taiwan consists of islands in East Asia, having an area of 35,808 square kilometers, with mountain ranges dominating the eastern two thirds and plains in the western third. With an estimated population of 23.76 million inhabitants, Taiwan is among the world’s most densely populated states. The Tropic of Cancer lies across the middle of Taiwan, giving Taiwan a humid subtropical climate in the north and central regions; a tropical monsoon climate in the south; and a temperate climate in the mountainous regions [7]. High temperature and humidity are island climate characteristics of Taiwan, which are conducive to the breeding of mosquitoes and the spread of mosquito-borne viruses [8]. The primary mosquito vector of Dengue virus in Taiwan is Aedes aegypti, followed by some transmission by Aedes albopictus [1]. In Taiwan, Aedes aegypti are distributed in south of the Tropic of Cancer, while Aedes albopictus are distributed throughout the island [9, 10]. The main epidemic areas of dengue fever are located in southern Taiwan, including Kaohsiung City, Tainan City, and Pingtung County [11–13]. In 2014 and 2015, southern Taiwan experienced the most serious dengue outbreak in recent years, with more than 40,000 dengue infected cases and more than 200 deaths [14]. Consequently, the Taiwan government established the National Mosquito-Borne Diseases Control Research Center (NMDC) under the National Health Research Institutes (NHRI) in 2016 to encourage research on the development of new tools or novel approaches to dengue epidemic control and to enhance research capacity on vector borne diseases.

In the past decade, studies have found evidence of a relationship between the climate and current weather conditions of a particular geographical area and the number of dengue cases [15, 16]. Various models have been developed to predict dengue outbreaks using climatic data. For example, Jing et al. used an autoregressive integrated moving average model incorporating external regressors to examine the association between the monthly number of locally acquired dengue infections and imported cases, mosquito densities, temperature and precipitation [17]. Although dengue-prediction models have been developed in many countries across the world, few studies have been reported in Taiwan [8, 18]. Wu et al. (2007) used the autoregressive integrated moving average model to analyze the monthly incidence of dengue fever in Kaohsiung, the largest city in southern Taiwan, from 1988 to 2003, and established a predictive model for forecasting the monthly incidence of dengue fever from 2004 to 2006 [8]. Yu et al. (2016) proposed an integration of the distributed lag nonlinear model (DLNM) and the Bayesian maximum entropy method to investigate the epidemics of dengue fever in southern Taiwan during 1998–2011, and developed a one-week-ahead dengue fever warning system. Yu et al’s approach could provide early warnings of dengue fever occurrences in 2012 and obtain the spatiotemporal distribution of dengue fever epidemics [18]. However, the data employed by the above methods were all from before 2012, and none of these analytic methods have been used to predict or investigate the outbreaks of 2014 and 2015. The approach proposed by Yu et al. is very complicated. The DLNM method uses functions (such as "poly" to generate the basis matrix of polynomials; and "crossbasis" to generate a cross-basis matrix of a DLNM) and parameters (such as "degree" to define the degree of a polynomials; and "argvar, arglag" to generate two basis matrices for predictor and lags, respectively) which are unintuitive and difficult to interpret, especially for non-statistical scientists, while our model is relatively easy to understand.

In this study, we apply a newly developed autoregressive model (AR model) with additional lagged weather predictors to examine the association between daily weather variability and daily dengue case number. For the 2014 and 2015 outbreaks of dengue fever, we developed 5-day-ahead and 15-day-ahead predictive models to predict the occurrence of dengue cases in Kaohsiung, the largest city in southern Taiwan. We hope that the proposed approach can be adopted by public health agencies in planning for dengue-control programs. The 5-day-ahead and 15-day-ahead predictions can provide early warning of the occurrence of dengue fever to assist public health agencies in preparing an epidemic response plan.

## Materials and methods

### Study area

Kaohsiung City is the third-largest city in Taiwan, with a land area of approximately 2,952 square km and a population of 2.77 million. Kaohsiung city is located south of the Tropic of Cancer. According to the Köppen climate classification [19], it belongs to a tropical monsoon climate, with high temperatures throughout the year and obvious dry and rainy seasons. The mean temperature ranges from 19.3 °C to 29.2 °C, with a mean high temperature of up to 32.4 °C and a mean relative humidity of 75.9% [20].

Kaohsiung was selected as the study area due to an abnormally increased incidence of dengue fever in 2014 and 2015 including 15,008 cases in Kaohsiung in 2014 and 19,710 cases in Kaohsiung in 2015. This is significantly higher than baseline, which encompassed approximately 300–2,000 cases annually in the preceding years, 2005–2013 [14, 21–25].

### Data collection

**Fig 1:**
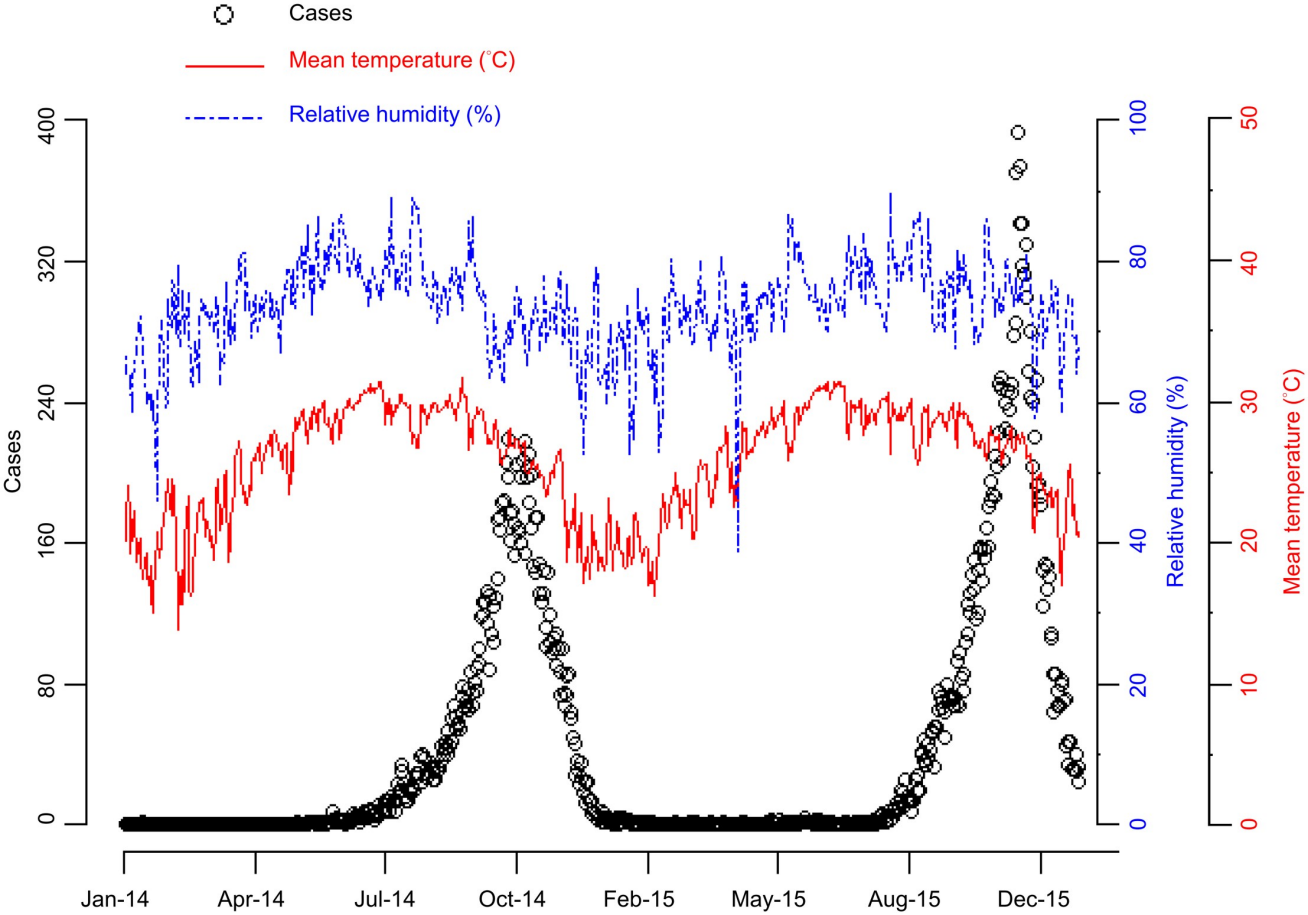
Trend plot of daily dengue case number and daily meteorological data for Kaohsiung City in 2014–2015. Dengue case number (open circles), mean temperature (red solid line), relative humidity (blue dashed line, scale at right).

The daily dengue case data for Kaohsiung City in 2014 and 2015 came from Taiwan’s Government Open Data Platform [26]. Meteorological data, including daily mean temperature and daily relative humidity, were obtained from the Central Weather Bureau [20]. Fig 1 shows the daily changes in the number of dengue cases and meteorological data during the study period in Kaohsiung City.

### Statistical analysis

**Fig 2:**
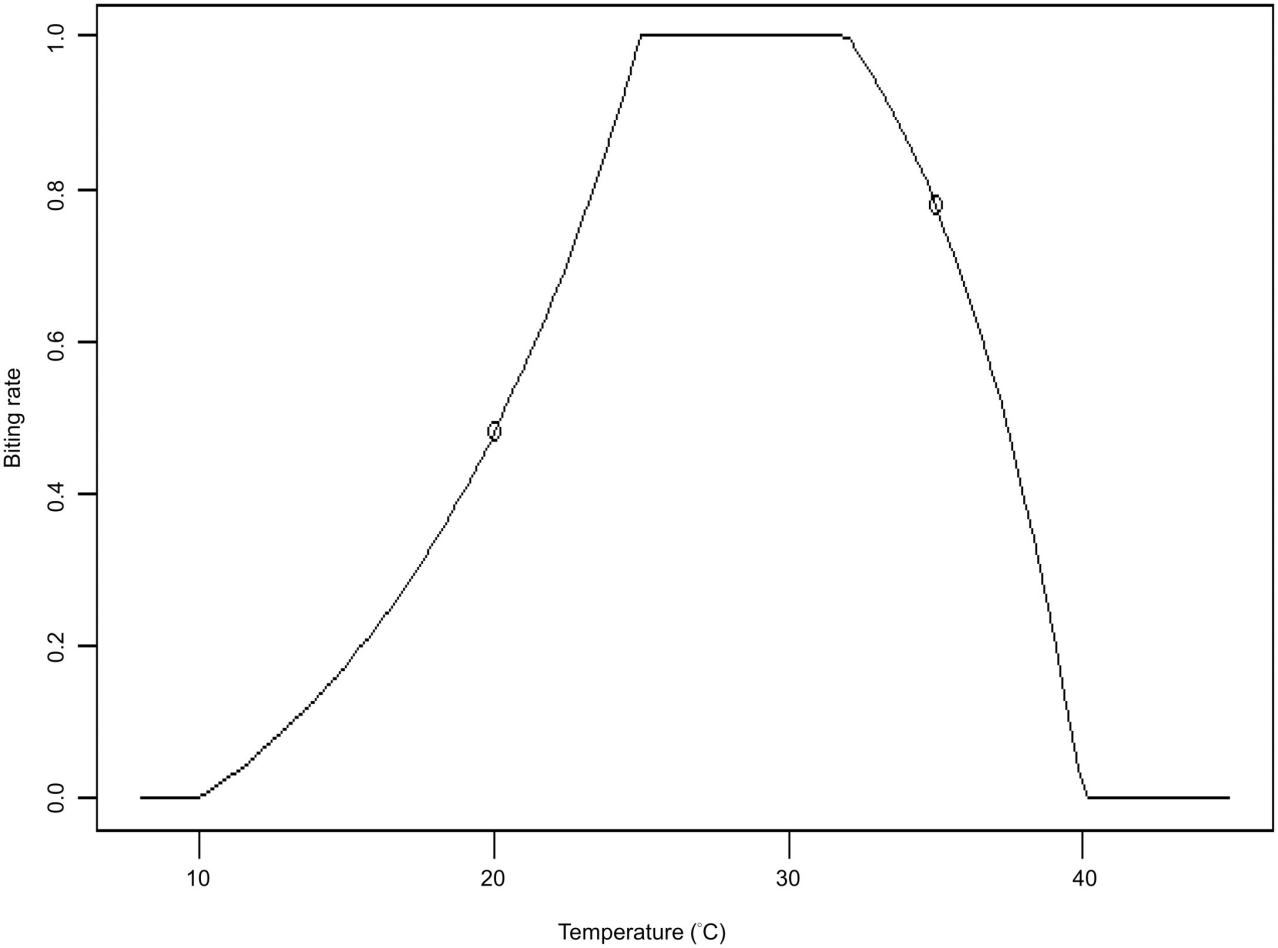
The relationship between temperature and the biting rate. The biting rate variable measures on a unit-less scale from 0 to 1 the likely proportion of the maximum mosquito biting-rate.

The relationship between temperature and mosquito activity has been reported in many published papers [21, 25, 27–30], motivating us to establish the mosquito biting rate function. The biting rate (BR) is a function of temperature and is used instead of temperature to explore the relationship with dengue case number. The known effect of temperature on mosquito activity is such that very low or high temperatures correspond to very low biting activity of mosquitos (Fig 2). The choice of constants and shapes in the biting rate function is based on some scientific literature [30, 31]. For example, for temperatures below 10 °C or temperatures above 40 °C, the biting rate is equal to zero; for temperatures in the range of 25–32 °C, the biting rate is in the middle plateau. We use biting rate function to capture the non-linear relationship between dengue transmission and temperature.

#### AR model 1 (M1)

The autoregressive model with additional lagged weather predictors was used to establish the relationship between dengue case numbers and potential risk factors, such as humidity, BR, and past counts of dengue cases. Suppose the current time point is t; we base our predictions on case and meteorological data at times preceding t up to a maximum lag of D days before t. The proposed autoregressive model is as follows:

$$Y_{t+k}=\alpha +\sum_{j=1}^{D}\beta_{j}Y_{t-j}+\sum_{j=1}^{D}\gamma_{j}H_{t-j}+\sum_{j=1}^{D}\delta_{j}{BR}_{t-j}+\varepsilon_{t+k},$$ (1)

where *Y*_*t*_ represents the case numbers at current time point t; *Y*_*t*+*k*_ represents the case numbers at time *t* + *k*; and k is the desired predict-ahead lag. In our settings *k* = 5 or 15 (5- or 15-day-ahead predictions); D is the maximum lag allowed in the model. The AR model based on Eq (1) is defined as model 1 (M1). The parameter *α* is the intercept term; *Y*_*t*−*j*_, *H*_*t*−*j*_, and *BR*_*t*−*j*_ are the number of past cases, the past humidity, and the past biting rate, with *β*_*j*_, *γ*_*j*_, and *δ*_*j*_ as their corresponding coefficients at lag j, respectively. The error terms *ε*_*t*+*k*_ are i.i.d. random variables with E(*ε*_*t*+*k*_|*F*_*t*−*j*_) = 0, where *F*_*t*−*j*_ = (*Y*_*t*−1_, …, *Y*_*t*−*D*_, *H*_*t*−1_, …, *H*_*t*−*D*_, *BR*_*t*−1_, …, *BR*_*t*−*D*_) are the past data. To estimate the parameters, the error terms *ε*_*t*+*k*_ are further assumed to be normally distributed. That is, *ε*_*t*+*k*_ ~ *N*(0, *σ*^2^), where *σ*^2^ is estimated by data. We chose D = 50 days, which means that we use the information of *H*_*t*−*j*_, *BR*_*t*−*j*_, and *Y*_*t*−*j*_ in time period [t-50, t-1] to predict the case number *Y*_*t*+*k*_. Based on the precedent set by Zeger & Qaqish (1988) and Slud & Kedem (1994), the parameter estimation is via a partial likelihood approach [32–34].

#### AR model 2 (M2)

We further proposed an autoregressive model, called M2 model, by inserting an interaction term between the predictor and the sign of the predictor minus some threshold (e.g., 100 cases). The specific model with a lagged interaction term can be written as:

$$Y_{t+k}=\alpha +\sum_{j=1}^{D}\beta_{j}Y_{t-j}+\sum_{j=1}^{D}\gamma_{j}H_{t-j}+\sum_{j=1}^{D}\delta_{j}{BR}_{t-j}+\sum_{j=1}^{D}\varphi_{j}Z_{t-j}+\varepsilon_{t+k},$$ (2)

where the interaction term at lag j is $Z_{t-j}=\{\begin{array}{c} \begin{array}{cc} 1*Y_{t-j} & ,if\;Y_{t-j}\geq 100\; \end{array}\\ \begin{array}{cc} -1*Y_{t-j} & ,\;if\;Y_{t-j}<100\; \end{array} \end{array}$, and *φ*_*j*_ is the corresponding coefficient. The interaction terms in this model showed major changes just before the peaks in numbers of Dengue cases. Thus, the M2 model might have the potential to markedly improve forecasting for recurrent infectious disease outbreaks such as dengue.

#### Model selection criteria

The selection criteria for the best-fit model are based on the Akaike information criterion (AIC), according to which smaller AIC values indicate better fit [8, 35–37]. AIC is defined as follows

$$AIC=-2\;\text{ln}\;(\hat{L})+2M,$$

where $\hat{L}\;$ is the maximum value of the likelihood function for the fitted model of fixed order *M*, where *M* is the number of model parameters. The first term on the right-hand side of the equation is a measure of the goodness of fit for the model; and the second term on the right-hand side of the equation is the penalty for redundant parameters to avoid overfitting. Another approach is given by G. Schwarz’s Bayesian Information Criterion (BIC) [38]. The formula for BIC is similar to the formula for AIC by replacing the 2M term (in the formula of AIC) by J*M, where J = log(sample size) is a scaling parameter controlling the number of terms in the model. Compared with AIC, BIC has a stronger penalty for redundant parameters. In this article, we also compared these two selection criteria.

The model was repeatedly refitted on data up through time *t*, and then the predictions at *t* +*k* were compared with actual outcomes.

#### Evaluation of model performance

For the selected models of type M1 and M2, we use the Pearson correlation coefficient, mean absolute error (MAE), and root mean square error (RMSE) to evaluate the performance of model prediction. The mean absolute error (MAE) and root mean square error (RMSE) are defined as follows:

$$MAE=\frac{\sum_{t\in v}\left|Y_{t+k}-{\hat{Y}}_{t+k}\right|}{n\;(v)}$$

$$RMSE=\sqrt{\frac{\sum_{t\in v}{\left(Y_{t+k}-{\hat{Y}}_{t+k}\right)}^{2}}{n\;(v)}}$$

where *Y*_*t*+*k*_ is the actual number of dengue cases *k* days after time *t* when the prediction is made, and ${\hat{Y}}_{t+k}$ is the number of cases forecasted by the model. The notation *v* is the validation set, and n (ν) denotes the number of time-points t in the validation set. In addition, we draw residual plots to compare the observed values with the predicted values of the fitted models to examine the goodness of fit for these models.

#### Sensitivity analysis

**Fig 3:**
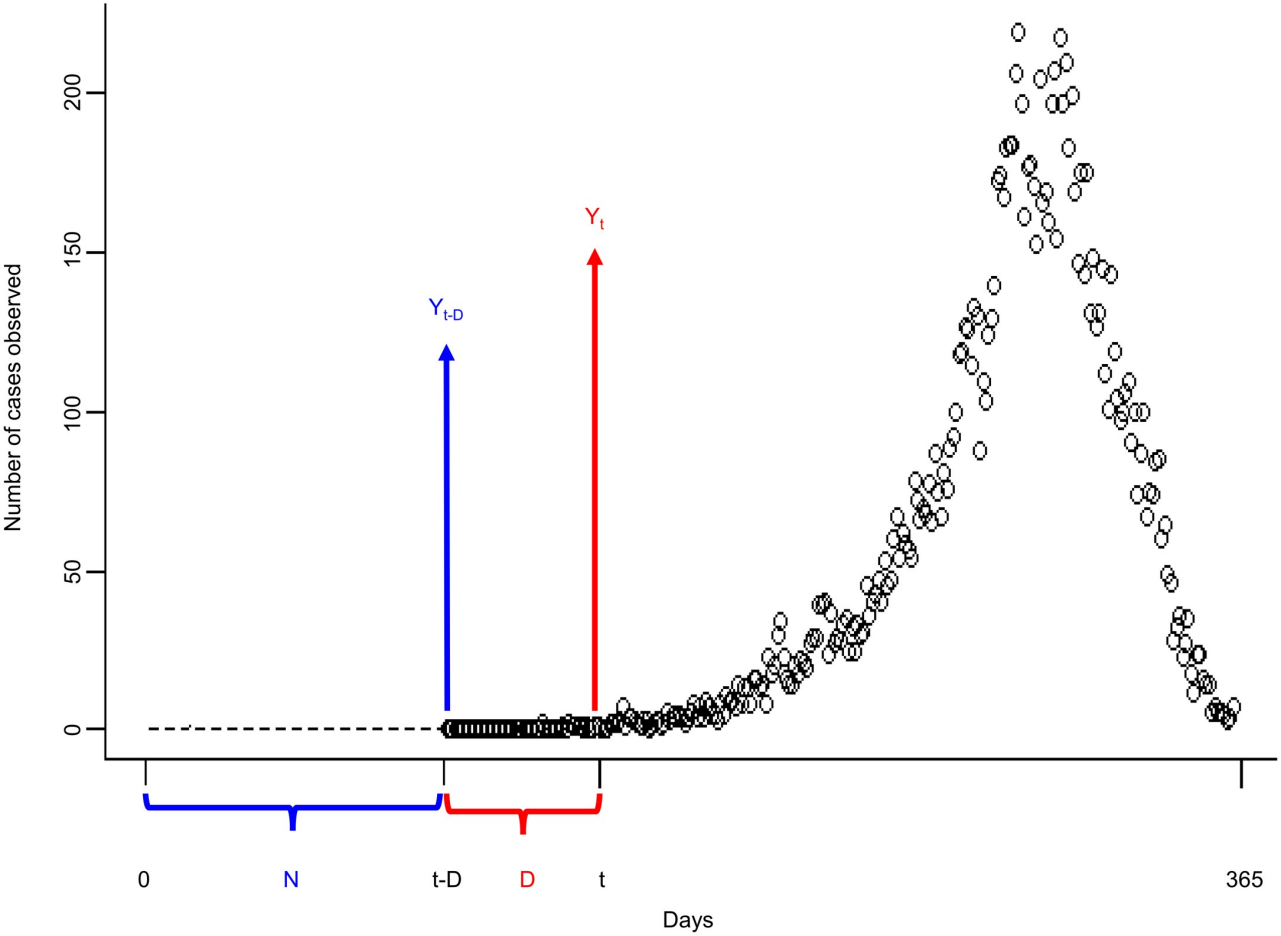
Schematic diagram of tuning parameters for sensitivity analysis. N is the number of values used for initialization, indicated by the dotted line. D is the maximum lag allowed in the model. The open circles represent the real dengue case counts Y_s_ at times s from t-D to 365 days.

Sensitivity analysis was used to assess the effects of tuning parameters. There are two tuning parameters in this study:

1. N: length of starting values. (Please see Fig 3); and
2. D: maximum lag in AR model. More specifically, D is the maximum lag allowed in the model (as shown in Fig 3).

We explore the effect of the changes in these two parameters on the prediction results.

#### Real-time dengue case forecast

The models we developed (M1 or M2) can be an integral part of Kaohsiung’s dengue control program. The final model can be embedded in a “real-time” schedule, with data (at present) being updated daily or weekly depending on the need of public health workers. In real-time forecast setting, the data were updated weekly (for example, updated on every Monday) and the real-time dengue case forecasts predicted the daily incidence of dengue notifications for both the 5-day and 15-day-ahead models. More specifically, we refit the models at the various time-points using only earlier data (from time-point t-D to the t). Variable selection was performed only once in a week (e.g., on Monday) and the predictions be made using the fixed current set of selected lagged variables until that set is updated. Real-time forecasts are for disease prevention or control, enabling public health agencies to take effective action to mitigate the potential risks of disease outbreaks and to implement vector-control programs.

All of these analyses were performed using R software (version 3.4.2).

## Results

### Comparison of predicted results with actual dengue case records

**Fig 4:**
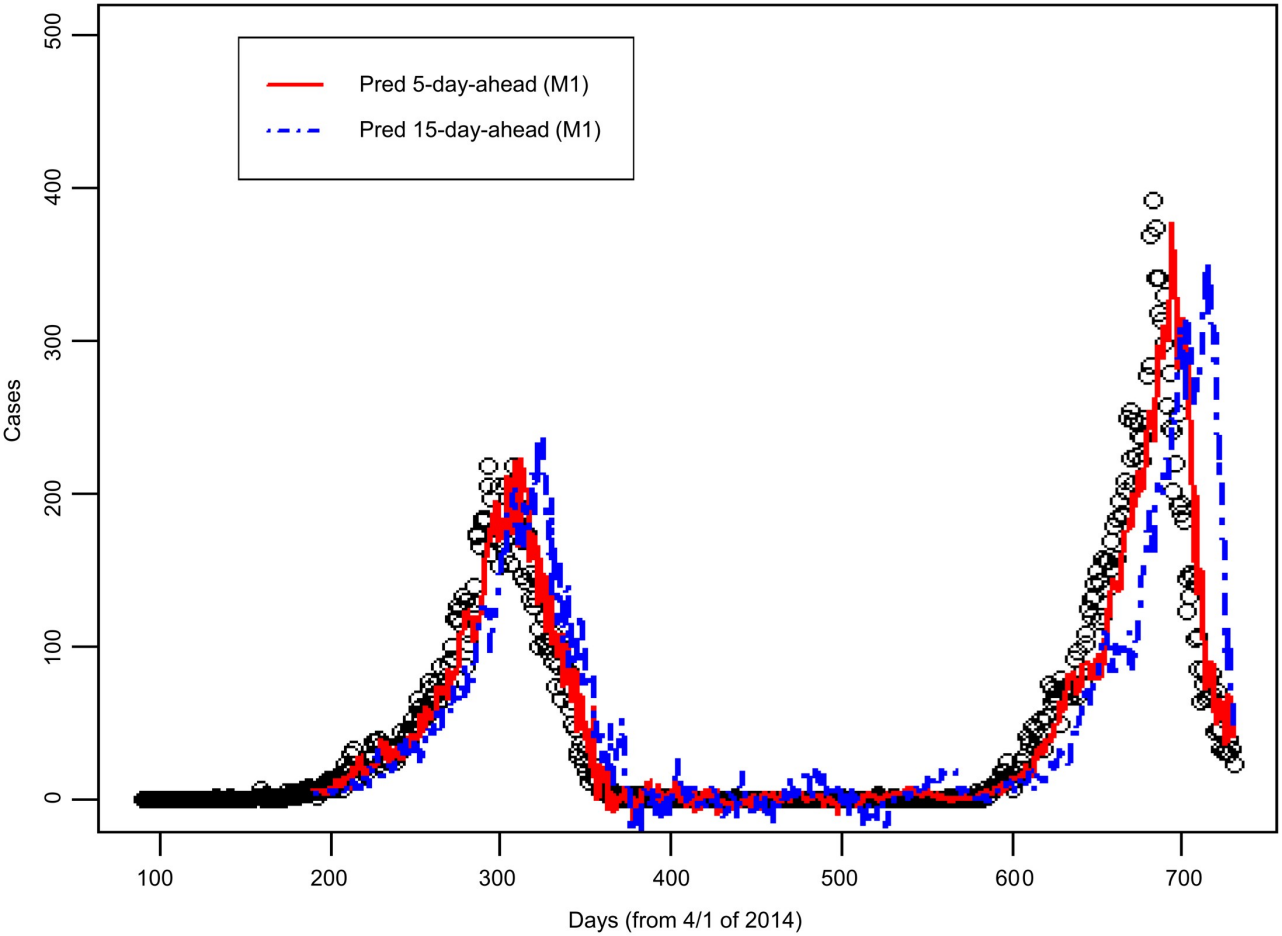
Comparison of actual dengue case records and predicted case number in Kaohsiung City from 2014 to 2015 based on the M1 model. (Open circles: Dengue case records; solid line: 5-day-ahead prediction; dashed line: 15-day-ahead prediction. “Pred 5-day-ahead” represents “Predicted 5 days ahead”. “Pred 15-day-ahead” represents “Predicted 15 days ahead”).

**Fig 5:**
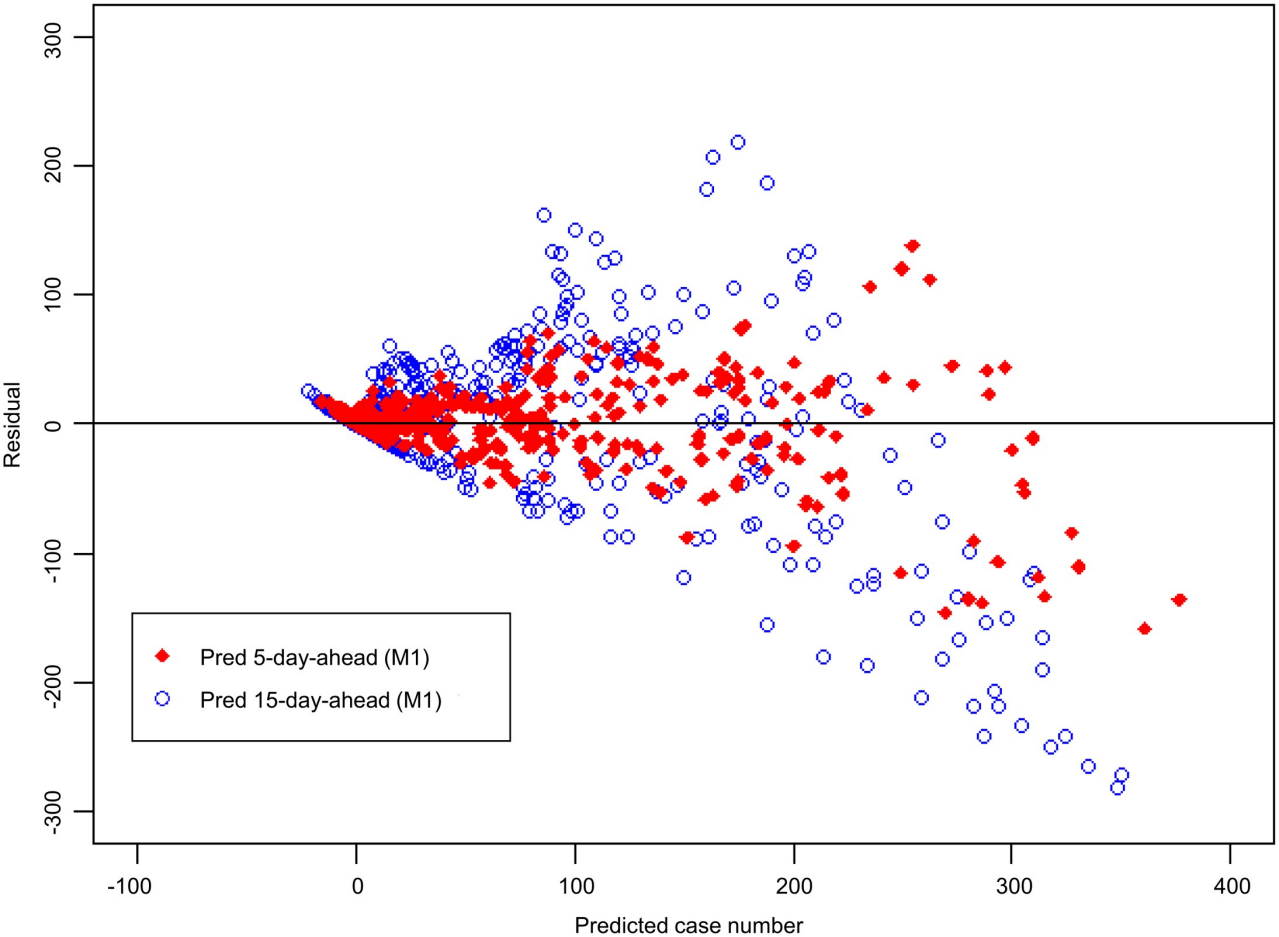
Residual plot corresponding to Fig 4. Y-axis: residual; X-axis: predicted number of dengue cases. “Pred 5-day-ahead” represents “Predicted 5 days ahead”. “Pred 15-day-ahead” represents “Predicted 15 days ahead”.

Fig 4 shows the number of dengue cases observed in Kaohsiung City from 2014 to 2015, as well as the 5-day-ahead and the 15-day-ahead dengue case number predicted using the M1 model. The figure shows that the peak predicted by the 5-day-ahead model was synchronized with the peak of actual dengue case number, and the peak predicted by the 15-day-ahead model was slightly delayed. The correlation coefficient between the dengue case number predicted by the 5-day-ahead model and the observed dengue case number was 0.93; the correlation coefficient between the dengue case number predicted by the 15-day-ahead model and the observed dengue case number was 0.71 (see M1 model in Table 1). The residual analysis is shown in Fig 5. The y-axis in Fig 5 represents the residual value, which is the difference between the actual and predicted numbers of cases. A value of the y-axis less than zero indicates that the actual number of cases is smaller than the corresponding predicted number. The variability of residual values increases with the number of predicted dengue cases. A possibly effective way to handle this would be to use the M2 model.

**Table 1:** Comparison between actual dengue case number and predicted dengue case number.

	2014–2015 without interaction terms (M1)		2014–2015 with interaction terms (M2)	
	5-day-ahead	15-day-ahead	5-day-ahead	15-day-ahead
Pearson’s correlation	0.93	0.71	0.95	0.82
MAE	16.2	35.5	14.7	29.1
RMSE	29.3	60.8	25.5	47.1

### Comparison between M1 model and M2 model

**Fig 6:**
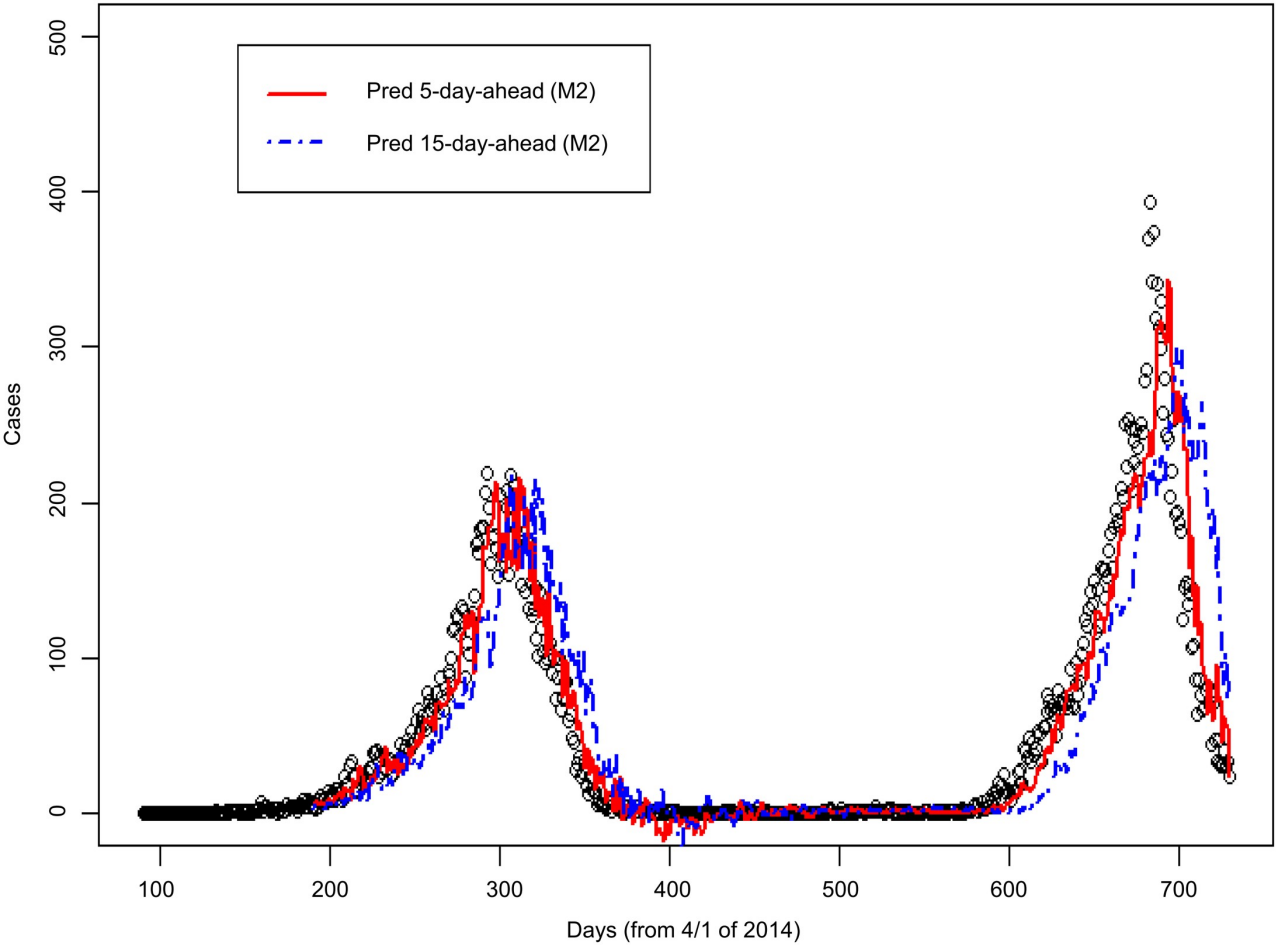
Comparison of actual dengue case records and predicted case number in Kaohsiung City from 2014 to 2015 based on the M2 model. (Open circles: Dengue case records; solid line: 5-day-ahead prediction; dashed line: 15-day-ahead prediction. “Pred 5-day-ahead” represents “Predicted 5 days ahead”. “Pred 15-day-ahead” represents “Predicted 15 days ahead”).

**Fig 7:**
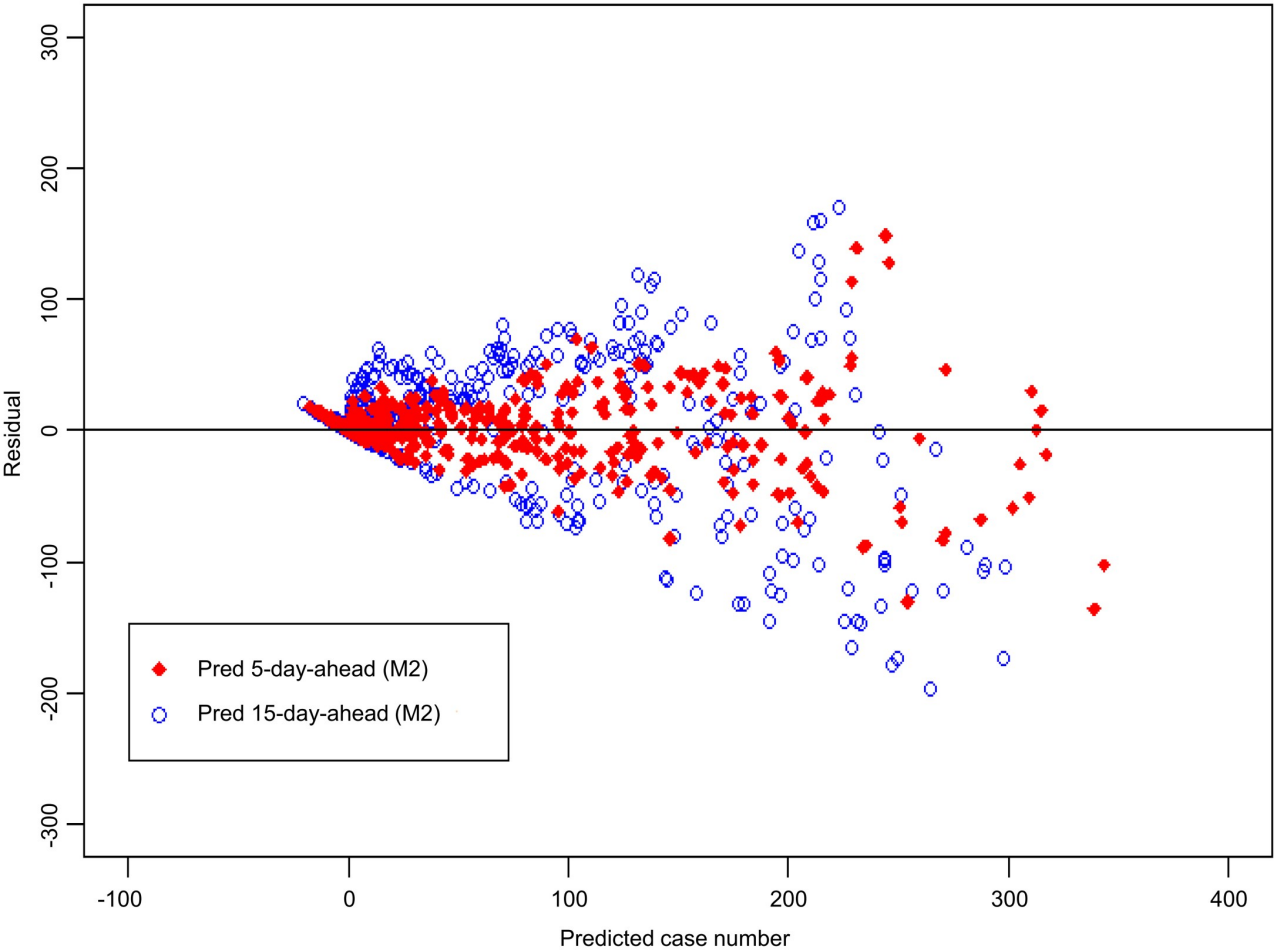
Residual plot corresponding to Fig 6. Y-axis: residual; X-axis: predicted number of dengue cases. “Pred 5-day-ahead” represents “Predicted 5 days ahead”. “Pred 15-day-ahead” represents “Predicted 15 days ahead”.

The results of using the M2 model are presented in Figs 6 and 7. According to the residual plots of M1 (Fig 5) and M2 (Fig 7), the fitted case number of M2 model is better than that of M1 model. Table 1 shows that Pearson’s correlation coefficient increased from 0.71 in the M1 model to 0.82 in the M2 model. Table 1 also shows that the MAE is reduced from 35.5 in the M1 model to 29.1 in the M2 model; the RMSE is reduced from 60.8 in the M1 model to 47.1 in the M2 model. Under the various evaluations described above, the predictive performance of the M2 model outperforms the M1 model.

### Prediction results for models built using 2014 and 2015 data, respectively

The 2015 dengue outbreak started relatively late compared to the 2014 outbreak, and the peak of dengue case counts was higher and narrower in 2015 than in 2014 (see Fig 1). Due to the slightly different temporal patterns of dengue outbreaks in 2014 and 2015, we separate the 2014 and 2015 data to establish models for 2014 and 2015, and predict dengue case number in 2014 and 2015. For more details about the model predictions for the 2014 and 2015 data, please refer to S1 and S2 Figs (S1 Fig is the results of 2014; S2 Fig is the results of 2015).

### Comparison between AIC and BIC

Table 2 shows a comparison of the models selected using the AIC or BIC selection criteria. From the comparison of correlation coefficient, MAE and RMSE, the performance of predictive models selected by AIC or BIC criteria is essentially the same. For example, in the M1 model of 2014 with a 5-day-ahead prediction, the correlation between the predicted values and the actual values of the model is 0.93 when either the AIC or BIC selection is used. According to Bozdogan [39, 40], AIC lacks certain properties of asymptotic consistency. Although BIC takes a similar form like AIC, it is derived within a Bayesian framework, reflects sample size and have properties of asymptotic consistency. Thus, we suggest to use BIC criterion for model selection.

**Table 2:** Comparison of AIC and BIC model selection criteria.

		AIC		BIC	
		5-day-ahead	15-day-ahead	5-day-ahead	15-day-ahead
Pearson’s correlation	2014 M1 model	0.93	0.76	0.93	0.76
	2014 M2 model	0.93	0.79	0.93	0.79
	2015 M1 model	0.94	0.74	0.93	0.73
	2015 M2 model	0.94	0.76	0.94	0.76
MAE	2014 M1 model	16.9	33.1	17.0	33.1
	2014 M2 model	16.3	31.3	16.2	31.4
	2015 M1 model	22.7	52.5	22.9	52.7
	2015 M2 model	22.0	49.1	22.2	49.2
RMSE	2014 M1 model	23.0	44.3	22.9	44.3
	2014 M2 model	22.3	40.6	22.3	40.6
	2015 M1 model	36.5	77.0	36.0	76.1
	2015 M2 model	34.6	70.3	34.5	70.3

### Sensitivity analysis

In this section, we perform univariate and bivariate sensitivity analyses to evaluate how the robustness of the model is affected by two tuning parameters: N (length of starting values) and D (maximum lag). In the univariate sensitivity analysis, the parameter we first evaluated was N. We let D be a fixed constant, such as 50, and allow N to vary between 90 and 120. Second, we evaluate the sensitivity analysis of the next parameter, D. We let N be a fixed constant, such as 100, and allow D to vary between 40 and 60. Finally, we evaluate the bivariate sensitivity analysis. In other words, we examine whether the estimates of the prediction results are robust with simultaneous changes in N and D.

S1 Table shows the results of the sensitivity analysis. Overall, small variations in N and D do not produce large changes in the results of interest, suggesting that the results observed in the main model (N = 100 & D = 50) are robust in this analysis.

### Real-time dengue forecast

**Fig 8:**
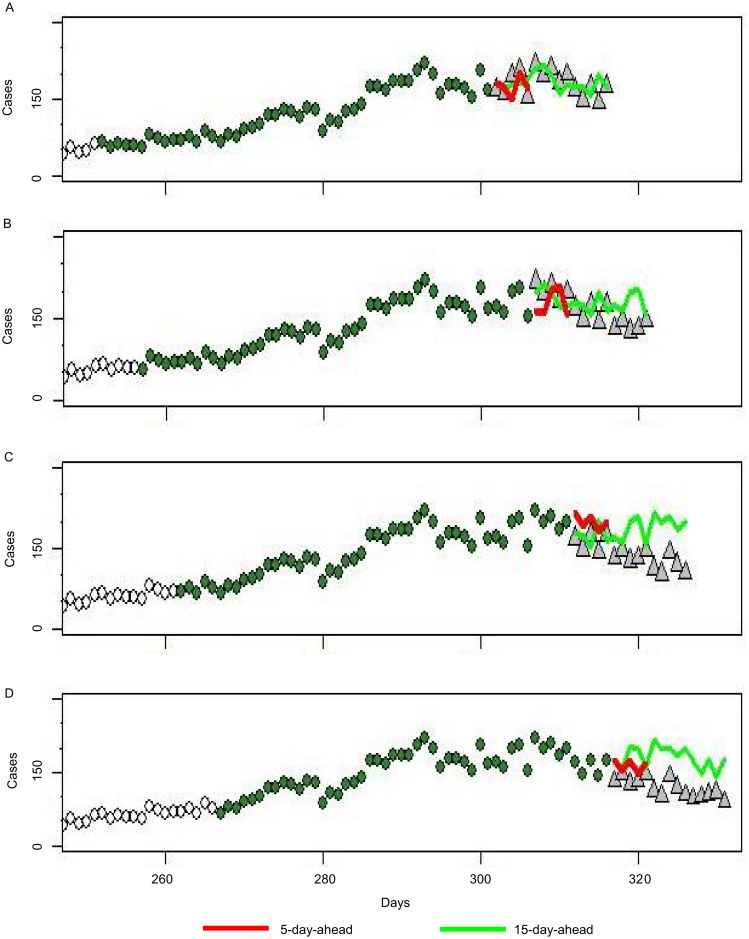
Real-time dengue case forecast in Kaohsiung City in 2014 based on the M2 model. In the real-time test of the model for the 2014 outbreak, real data that existed on four different start dates were inputted: (A) October 29, (B) November 3, (C) November 8 and (D) November 14. (Open circles: Dengue case records; green circles: data in D; grey triangles: actual value at prediction time).

**Fig 9:**
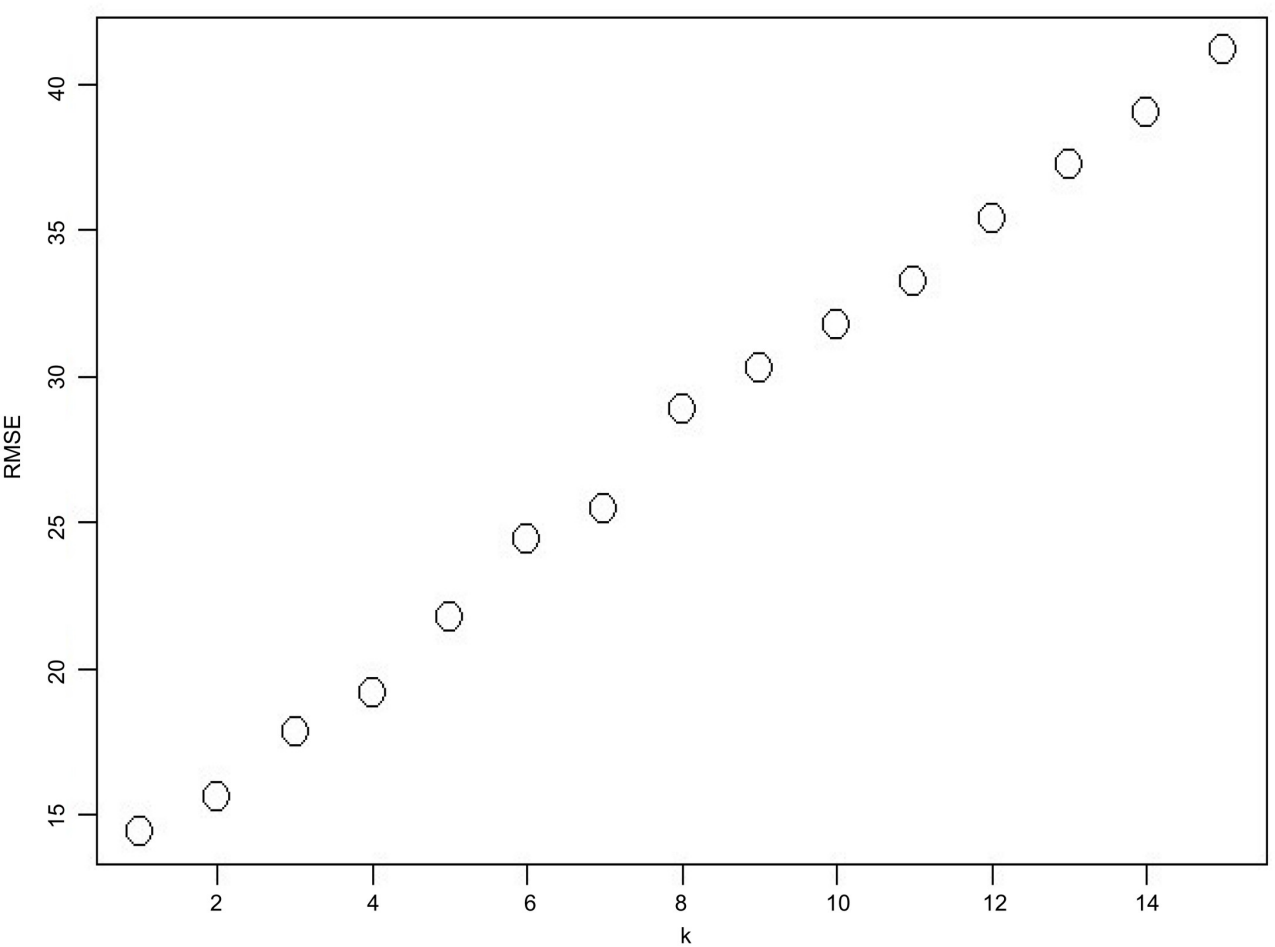
The relationship between RMSE and k.

Fig 8 shows the forecasts of different time points for the 2014 dengue fever epidemic in Kaohsiung. Starting dates for prediction are October 29, November 3, November 8, and November 14 for Fig 8A, 8B, 8C and 8D, respectively. The prediction of the 5-day-ahead model is synchronized with the actual data. In the prediction of the 15-day-ahead model, the end of the epidemic was relatively late and the scale of the epidemic was relatively large. Overall, the 5-day-ahead model produces more accurate predictions than the 15-day-ahead model. Fig 9 shows the relationship between RMSE and k. When k is large, the RMSE is large, which indicates poor prediction performance. When we are doing real-time forecast, we can further improve the model. For the 5-day-ahead model, we can use k = 1 to predict the first day, k = 2 to predict the second day, k = 3 to predict the third day, and then to k = 5 to predict the fifth day. This will be more accurate than using k = 5 for all predictions.

## Discussion

In this study, we assessed whether meteorological factors affected the dengue epidemic in Kaohsiung City, southern Taiwan. We developed 5-day-ahead and 15-day-ahead predictive models. Data on dengue case number and weather variables from 2014 to 2015 were used in model analysis. Our research showed that humidity and biting rate (BR) were strongly associated with dengue case counts in Kaohsiung City. Previous studies have shown that weather factors such as temperature, humidity, and rainfall may affect dengue transmission [37, 41]. However, in our study, rainfall was not significant for predicting the occurrence of dengue cases for years 2014 and 2015. This result is consistent with the findings of Wu et al. [8]. According Yu et al [18], their study showed dengue fever risk increases as soon as weekly maximum 24-h rainfall exceeds approximately 50 mm. If the time of rainfall and last for at least 3 months, the risk for dengue fever can increases. The rainy days with exceed 50 mm/day just have eleven days and ten days in 2014 and 2015, respectively, for Kaohsiung city. Hence, the impact of rainfall in Kaohsiung City was very limited and we guessed the reason was precipitation/rainfall typically observed in Kaohsiung City usually was under the threshold. This may also be related to the fact that Aedes aegypti prefer to inhabit indoors, so the impact of rainfall may be limited [42].

The approach we proposed has many advantages for government agencies fighting dengue epidemics. First, the model is simple, intuitive, and easy to use. When dengue outbreaks suddenly occur, the model timely is possible to predict outbreaks and to prevent them. If the model is simple, then a single trained individual is required to input the variables daily or weekly. If the model is complicated, then we need a people trained by a lot of profession class and it is hard to extend this model. Second, the modeling time in computing is fast. Third, adaptive model prediction can be achieved by continuously updating and accumulating information over time. That is, the default value of D is 50. When more data are accumulated, D can be increased. We can increase D to be 60 or 70 for using more data as predict. Finally, our real-time forecast model using adaptive model prediction can meet the local government’s epidemic prevention needs. However, this study has some limitations. First, the models we constructed were for predictive accuracy, but they were not suitable to explain the cause of the outbreak. The proposed model is not able to explain the specific reason for the outbreak due to a lack of adjustment for other factors, such as seasonal information or a combination of all available weather factors. In this model, we allow the effects of each factor (such as humidity) to have multiple lags in affecting future dengue case number, so interpretation of meteorological factors becomes difficult. Second, in this study, we included only meteorological variables but did not include data on mosquito populations, such as house index (HI), container index (CI), and Breteau index (BI). We have not considered the impact of imported cases and cluster infections. Finally, the accuracy of our model may be offset by disease-control interventions undertaken by local governments. Despite the above limitations, simple models that use only climatic factors perform well in the circumstances described. The results of the study can be provided to the local health departments for reference. Future research can consider including a combination of all available weather factors in the model (e.g., seasonal information) based on longer meteorological data records, creating some interaction terms between key predictor terms such as rainfall, temperature, and humidity.

In this study, we applied a newly developed autoregressive model (AR model) with additional lagged weather predictors to establish 5-day-ahead and 15-day-ahead predictive models to predict the occurrence of dengue cases. Although the prediction accuracy decreases as the prediction period increases (e.g., from 5 days ahead to 15 days ahead), this inaccuracy is still tolerable. The correlation between the predicted values estimated by 5-day model and the actual values is 0.9 which is high. The correlation estimated by 15-day model is 0.7 which is still tolerable. We hope that the proposed real-time forecast model can serve as a reference for government agencies fighting dengue epidemics.

## Supporting information

S1 Fig Comparison of actual dengue case records and predicted case number in Kaohsiung City based on M1 model and M2 models. Only the 2014 data were used to construct the model.
(DOCX)

S2 Fig Comparison of actual dengue case records and predicted case number in Kaohsiung City based on M1 and M2 models. Only the 2015 data were used to construct the model.
(DOCX)

S1 Table Sensitivity analysis. (DOCX)
