# Supplementary material for: Real-time dengue forecast for outbreak alerts in Southern Taiwan
Source: PLoS Negl Trop Dis. 2020 Jul 27;14(7):e0008434. doi: 10.1371/journal.pntd.0008434 (PMC7384612; doi:10.1371/journal.pntd.0008434)
Supplement: S1 Fig — Only the 2014 data were used to construct the model. (DOCX) [file pntd.0008434.s001.docx]

**S1 Fig.** **Comparison of actual dengue case records and predicted case number in Kaohsiung City based on M1 model and M2 models.** Only the 2014 data were used to construct the model.

1. Kaohsiung City's actual dengue case records in 2014 and predicted case number based on M1 and M2 models. (Open circles: Dengue case records; solid line: M1 model; dashed line: M2 model. “Pred 5-day-ahead” represents “Predicted 5 days ahead”. “Pred 15-day-ahead” represents “Predicted 15 days ahead”.)


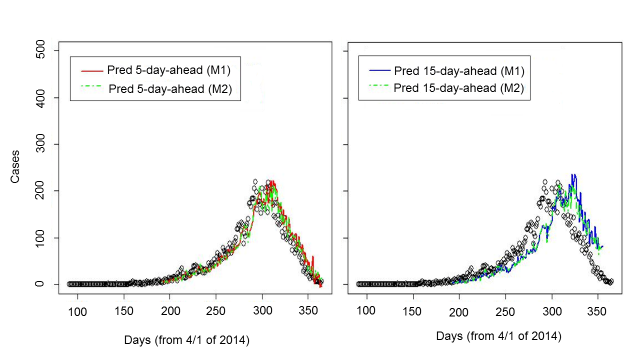


(B) Residual plot corresponding to Supplementary figure 1 (A). Y-axis: residual; X-axis: predicted number of dengue cases. “Pred 5-day-ahead” represents “Predicted 5 days ahead”. “Pred 15-day-ahead” represents “Predicted 15 days ahead”.


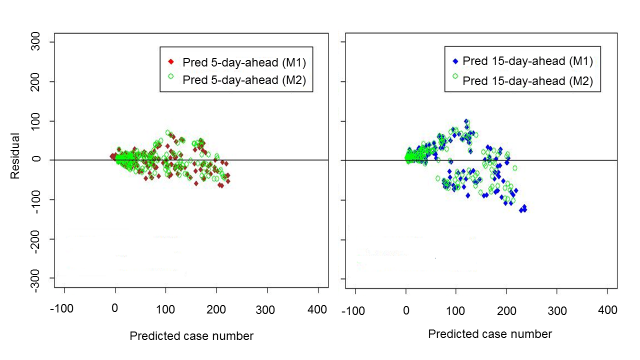


(C) Evaluation of model performance. Only the 2014 data were used to construct the model.

|  | 2014 without interaction terms (M1) | |  | 2014 with interaction terms (M2) | |
| --- | --- | --- | --- | --- | --- |
|  | 5-day-ahead | 15-day-ahead |  | 5-day-ahead | 15-day-ahead |
| Pearson’s correlation | 0.93 | 0.76 |  | 0.93 | 0.79 |
| MAE | 16.9 | 33.1 |  | 16.3 | 31.3 |
| RMSE | 22.9 | 44.3 |  | 22.3 | 40.6 |
