# Supplementary material for: Real-time dengue forecast for outbreak alerts in Southern Taiwan
Source: PLoS Negl Trop Dis. 2020 Jul 27;14(7):e0008434. doi: 10.1371/journal.pntd.0008434 (PMC7384612; doi:10.1371/journal.pntd.0008434)
Supplement: S1 Table — (DOCX) [file pntd.0008434.s003.docx]

**S1 Table. Sensitivity analysis.**

1. Pearson’s correlation coefficient

|  |  | 2014 | |  | 2015 | |
| --- | --- | --- | --- | --- | --- | --- |
| Length of starting values (N) | Maximum lag (D) | 5-day-ahead | 15-day-ahead |  | 5-day-ahead | 15-day-ahead |
| 90 | 40 | 0.93 | 0.73 |  | 0.93 | 0.67 |
|  | 50 | 0.93 | 0.73 |  | 0.93 | 0.68 |
|  | 60 | 0.93 | 0.73 |  | 0.93 | 0.68 |
| 100 | 40 | 0.93 | 0.73 |  | 0.93 | 0.68 |
|  | 50 | 0.93 | 0.73 |  | 0.93 | 0.68 |
|  | 60 | 0.93 | 0.73 |  | 0.93 | 0.68 |
| 110 | 40 | 0.93 | 0.73 |  | 0.93 | 0.68 |
|  | 50 | 0.93 | 0.73 |  | 0.93 | 0.68 |
|  | 60 | 0.93 | 0.73 |  | 0.93 | 0.68 |
| 120 | 40 | 0.93 | 0.73 |  | 0.93 | 0.69 |
|  | 50 | 0.93 | 0.73 |  | 0.93 | 0.69 |
|  | 60 | 0.93 | 0.73 |  | 0.93 | 0.69 |

1. MAE

|  |  | 2014 | |  | 2015 | |
| --- | --- | --- | --- | --- | --- | --- |
| Length of starting values (N) | Maximum lag (D) | 5-day-ahead | 15-day-ahead |  | 5-day-ahead | 15-day-ahead |
| 90 | 40 | 17.6 | 36.6 |  | 24.2 | 59.6 |
|  | 50 | 17.8 | 36.2 |  | 24.3 | 59.3 |
|  | 60 | 17.9 | 36.0 |  | 24.5 | 58.9 |
| 100 | 40 | 17.8 | 36.3 |  | 24.3 | 59.0 |
|  | 50 | 17.9 | 36.2 |  | 24.3 | 58.8 |
|  | 60 | 17.9 | 36.0 |  | 24.4 | 58.6 |
| 110 | 40 | 17.8 | 36.1 |  | 24.3 | 58.7 |
|  | 50 | 17.8 | 36.0 |  | 24.2 | 58.6 |
|  | 60 | 17.8 | 36.0 |  | 24.3 | 58.4 |
| 120 | 40 | 17.6 | 36.0 |  | 24.1 | 57.9 |
|  | 50 | 17.7 | 36.0 |  | 24.0 | 57.7 |
|  | 60 | 17.7 | 35.9 |  | 24.0 | 57.5 |

(C) RMSE

|  |  | 2014 | |  | 2015 | |
| --- | --- | --- | --- | --- | --- | --- |
| Length of starting values (N) | Maximum lag (D) | 5-day-ahead | 15-day-ahead |  | 5-day-ahead | 15-day-ahead |
| 90 | 40 | 23.3 | 48.4 |  | 39.4 | 85.2 |
|  | 50 | 23.6 | 47.5 |  | 38.9 | 84.4 |
|  | 60 | 23.7 | 47.0 |  | 38.8 | 83.7 |
| 100 | 40 | 23.6 | 47.5 |  | 38.7 | 84.1 |
|  | 50 | 23.6 | 47.2 |  | 38.5 | 83.7 |
|  | 60 | 23.6 | 47.0 |  | 38.5 | 83.2 |
| 110 | 40 | 23.5 | 47.2 |  | 38.5 | 83.5 |
|  | 50 | 23.6 | 47.0 |  | 38.3 | 83.3 |
|  | 60 | 23.5 | 46.8 |  | 38.3 | 83.0 |
| 120 | 40 | 23.4 | 46.9 |  | 38.0 | 82.0 |
|  | 50 | 23.5 | 46.8 |  | 38.0 | 81.7 |
|  | 60 | 23.5 | 46.7 |  | 37.9 | 81.4 |
